# Whole genome characterisation of Australian and New Zealand OsHV-1 ‘μVar’ like variants over time

**DOI:** 10.1016/j.virusres.2026.199798

**Published:** 2026-09-09

**Authors:** James O’Dwyer, Lachlan Coff, David Cummins, Peter G. Mohr, Nicholas J. Moody

**Affiliations:** CSIRO Australian Centre for Disease Preparedness, East Geelong, Victoria, 3220, Australia

**Keywords:** Genomics, Ostreid herpesvirus, Phylogeny, Diversity, Australia

## Abstract

Ostreid herpesvirus 1 (OsHV-1) is a major pathogen of Pacific oysters (*Magallana gigas*), linked to mass mortality events worldwide and presents a substantial threat to global aquaculture. Since 2010, mortality events have been occurring in Pacific oysters within Australia with the cause being an OsHV-1 microvariant highly similar to OsHV-1 μVar first detected in France, 2008. In this study, fifteen OsHV-1 whole genomes were assembled from Australian and New Zealand mortality events, including twelve newly sequenced genomes and three assembled from public datasets. Whole-genome phylogenetic analyses indicate that Australian and New Zealand OsHV-1 isolates form a distinct clade, separate from European and East Asian genomes, and with moderate regional and temporal divergence across the past 14 years. Across the dataset, multiple biologically relevant mutations were observed in genes of known function, including DNA polymerase, ribonucleotide reductase subunits, and apoptosis-related proteins, though most mutations occurred in uncharacterised genes. These findings highlight the limitations of single-gene typing and support the need for whole-genome approaches in understanding OsHV-1 evolution. Critically, the lack of recent whole-genome data, particularly from East and Southeast Asia, restricts the ability to trace viral origins and detect novel variants, underscoring the need for expanded global OsHV-1 genomic surveillance.

## Highlights

• Fifteen OsHV-1 µVar-like genomes assembled from Australia and New Zealand.
• Whole-genome phylogeny places Australia/New Zealand viruses in a distinct clade.
• Shared 6.5 kb deletion and recurrent indels concentrate in repeat-rich regions.
• Marker gene trees failed to detect clade divergence, supporting genome-wide typing and surveillance.

## 1 Introduction

Oyster cultivation dominates molluscan aquaculture production, and pacific oysters (*Magallana gigas*) make up a significant proportion, with a reported live weight production of 531 kt in 2023 (FAO, 2023, 2018). Pacific oyster mortality events pose a significant threat to oyster cultivation, with the capacity to impact global aquacultural production due to significant declines in oyster production. Ostreid herpesvirus 1 (OsHV-1) has consistently been identified as the primary cause of known mortality events of Pacific oysters (Fuhrmann et al., 2019). However, the mortality rate observed in individual outbreaks tends to be highly variable, with additional pathogenic agents such as *Vibrio* species, hatchery practices, and environmental conditions as potential contributing factors (Alfaro et al., 2019; De Decker et al., 2011; De Lorgeril et al., 2018; Huang et al., 2026; Keeling et al., 2014; Pernet et al., 2012; Segarra et al., 2010). However, the largest observed contributor to the severity of mortality events is the microvariant of OsHV-1 (EFSA, 2015). In 2008, a significant uptick in Pacific oyster mortality events coincided with the emergence of OsHV-1 microvariant (OsHV-1 μVar) in Europe, with reports of 80–100% mortality in French oyster populations attributed to this variant (EFSA, 2015). OsHV-1 remains a significant management challenge for oyster farmers worldwide, with the virus being detected in Europe, Asia, the Asia Pacific, Oceania, North, Central, and South America (Bai C et al., 2016; Barbieri et al., 2019; Barbosa Solomieu et al., 2015; Burge et al., 2021; Grijalva-Chon et al., 2013; Martenot et al., 2012, 2011; Sanjuán and Domingo-Calap, 2016; Shimahara et al., 2012).

OsHV-1 (*Ostreavirus ostreidmalaco1)* is a double-stranded DNA virus and the sole member of the genus *Ostreavirus* within the family *Malacoherpesviridae* (Davison et al., 2009; Varsani et al., 2024). The reference OsHV-1 genome is approximately 207 kbp in length, with the original complete genome sequence reported as 207,439 bp and predicted to encode 124 unique genes (Davison et al., 2005). The OsHV-1 reference genome exhibits an Arch-7 herpesvirus genome architecture, comprising two unique regions, U_L_ and U_S_, flanked by terminal and internal inverted repeats (TR_L_/IR_L_ and TR_S_/IR_S_), with an additional unique X region positioned between IR_L_ and IR_S_ (Davison et al., 2005; Dotto-Maurel et al., 2025). Despite the perceived genomic stability of DNA viruses, and particularly of herpesviruses, an abundance of variants of OsHV-1 have been discovered over the past two decades (Fuhrmann et al., 2019). OsHV-1 has been implicated in mortality events of various bivalve species, including the Manila clam (*Ruditapes philippinarum*), great scallop (*Pecten maximus***)**, European flat oyster (*Ostrea edulis***)**, and common cockle (*Cerastoderma edule*) (Arzul et al., 2001a, 2001b; Bookelaar et al., 2020; Morga et al., 2021; Hine et al., 1998). Some variants of OsHV-1, including OsHV-1-SB, OsHV-1 SW6 or acute viral necrosis virus (AVNV), have been observed to exclusively infect a novel host species (Bai et al., 2016; Morga et al., 2021; Ren et al., 2013), while the host species infected has been shown to also influence the genetic divergence and mutation rate of specific variants (Pelletier et al., 2025a).

Other variants observed after the classification of OsHV-1 μVar from the 2008 mortality outbreak in France have been associated with different outbreaks in oyster hatcheries including (but not limited to) OsHV-1 μVar Δ9, OsHV-1 µVar Δ15, OsHV-1 μVar B, and OsHV-1 μVar A (Burioli et al., 2017; Martenot et al., 2011). As the initial OsHV-1 μVar was shown to differ genetically from the original OsHV-1 genome (1995) in the microsatellite region within the gene ORF4, subsequent microvariants have been classified by mutations within this region (Segarra et al., 2010).

However, this approach overlooks significant genomic changes across the genomes of OsHV-1 taken from different regions (e.g., deletion-prone regions and substitution-rich regions (Morga et al., 2021), genes involved in host interactions, DNA replication, and membrane-associated functions (Pelletier et al., 2025a), and general whole genomic diversity (Bai et al., 2019), potentially masking phylogeographic structuring and distinct natural selection pressures outside of the target marker region. Thus, comprehensive genomic studies are essential for understanding the full spectrum of OsHV-1 variants and their implications for host–pathogen interactions.

Within Australia, the first recorded instance of a mortality event in Pacific oysters driven by OsHV-1 was observed in 2010 in the Georges River (New South Wales), in which the sequenced virus was found to be most similar to the OsHV-1 μVar variant, sharing the same microsatellite deletion which characterises μVar, resulting in the observed OsHV-1 being described as a μVar-like variant (Jenkins et al., 2013). Additional mortality events linked to OsHV-1 were observed in 2013 (Hawkesbury river, New South Wales), 2016 (Pitt Water, Tasmania), and 2018 (Port River, South Australia) (de Kantzow et al., 2017; Paul-Pont et al., 2014; PIRSA, 2018). In New Zealand, retrospective analyses indicate that OsHV-1 was already present in mortality-associated Pacific oyster material by 2005, but the first recorded outbreak in the literature occurred during the 2010–2011 summer mortality event (Keeling et al., 2014; Renault et al., 2012). During that outbreak, OsHV-1 PCR-negative spat transferred from a South Island hatchery into an OsHV-1-positive North Island farming area became rapidly infected, with 83% testing PCR-positive after 5 days and 100% after 7 days, while field mortalities became evident from Day 6 onward, demonstrating the rapid onset and high virulence of infection in naïve oyster spat under New Zealand field conditions (Keeling et al., 2014). The OsHV-1 μVar is the prevalent variant in both Australia and New Zealand, with slight variations observed in New Zealand isolates (Jenkins et al., 2013; Keeling et al., 2014). Recent studies have highlighted the variability in pathogenicity among OsHV-1 variants, both within Australia and in comparison to the initially identified OsHV-1 μVar variant detected in 2008 in France (Burge et al., 2020; Cain et al., 2021). However, these studies only sequenced the ORF4 gene, limiting understanding of how mutations across the whole genome may be driving pathogenicity changes (Burge et al., 2020; Cain et al., 2021).

While comprehensive genome-wide analyses are lacking, investigations into individual marker genes have revealed distinct nucleotide diversity and haplotypes unique to Australian OsHV-1 isolates when compared to those from other regions (Trancart et al., 2023). Furthermore, genetic polymorphisms have emerged as valuable tools for delineating geographic origins and discerning phenotypic differences, as demonstrated in vertebrate studies (Segarra et al., 2010). Here, we sequenced twelve full genomes of OsHV-1 from various Pacific oyster mortality events in Australia, dating back to 2010, and three full genomes from publicly available raw sequencing data for outbreaks in 2011 from New Zealand have been assembled. The purpose of this work is to determine the phylogenetic proximity of Australian and New Zealand samples to OsHV-1 μVar variants circulating in Europe and the Asia-Pacific, and to classify the emergence of any mutations in circulating Australian OsHV-1 genomes that may lead to expression changes in key genes associated with clinical signs or pathology in OsHV-1 infections.

## 2 Methods

### 2.1 Sample collection and DNA extraction

**Fig. 1:**
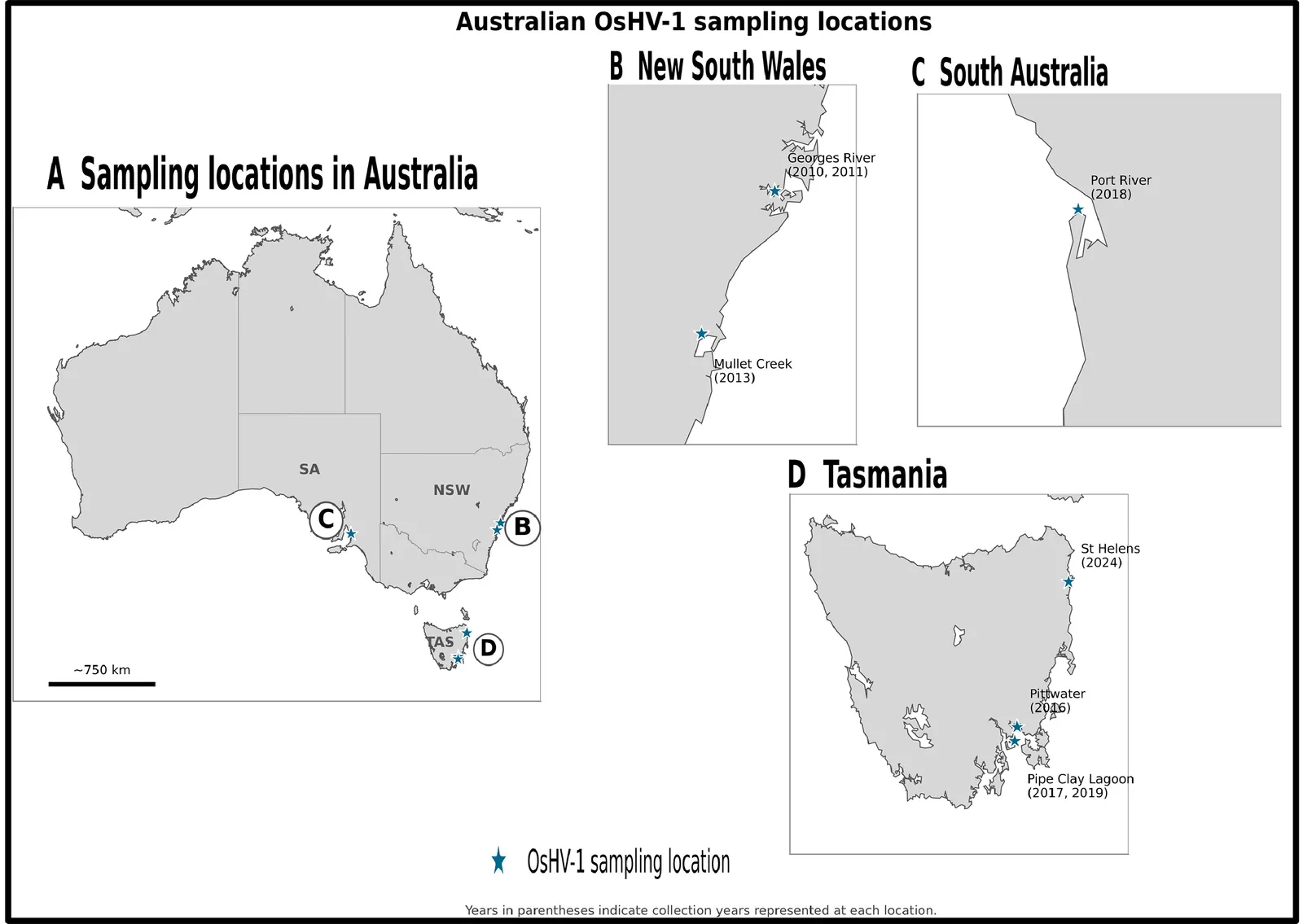
Collection sites of each sample. Sites are presented as approximate locations and not exact coordinates of collection.

Tissue of gills and mantle were collected from 12 samples of either farmed or wild Pacific oysters between the years 2010 and 2024 displaying symptoms of infection. Eleven samples of this tissue were sent directly to the Australian Centre for Disease Preparedness (ACDP) for DNA extraction and OsHV-1 detection, while one sample had DNA extracted and was sent to ACDP with no information on extraction method provided. For 11 samples, tissue was collected from a single individual, and one sample comprised multiple individuals collected in close proximity (Table 1, Fig. 1).

**Table 1:** Sample information and nucleic acid extraction kits used for each Australian sample in this study.

SAMPLE	YEAR	COLLECTION LOCATION	POOLED/SINGLE SAMPLE	EXTRACTION KIT
**OSHV_NSW_2010_1**	2010	Georges River, Sydney, NSW (farmed)	single	QIAamp DNA Mini Kit
**OSHV_NSW_2011_1**	2011	Georges River, Sydney, NSW (farmed)	single	Unknown
**OSHV_NSW_2013_1**	2013	Mullet Creek, Sydney, NSW (farmed)	single	QIAamp DNA Mini Kit
**OSHV_SA_2018_1**	2018	Port River, South Australia (wild)	single	MagMAX-96 Viral RNA Isolation Kit
**OSHV_TAS_2016_1**	2016	Pittwater, Tasmania (farmed)	single	QIAamp DNA Mini Kit
**OSHV_TAS_2016_2**	2016	Pittwater, Tasmania (farmed)	single	MagMAX-96 Viral RNA Isolation Kit
**OSHV_TAS_2018_1**	2017	Pipe Clay Lagoon, Tasmania (farmed)	single	MagMAX-96 Viral RNA Isolation Kit
**OSHV_TAS_2019_1**	2019	Pipe Clay Lagoon, Tasmania (farmed)	single	MagMAX-96 Viral RNA Isolation Kit
**OSHV_TAS_2019_2**	2019	Pipe Clay Lagoon & Pittwater, Tasmania (farmed)	pooled	MagMAX-96 Viral RNA Isolation Kit
**OSHV_TAS_2024_1**	2024	St. Helens, Tasmania (wild)	single	MagMAX-96 Viral RNA Isolation Kit
**OSHV_TAS_2024_2**	2024	St. Helens, Tasmania (wild)	single	MagMAX-96 Viral RNA Isolation Kit
**OSHV_TAS_2024_3**	2024	St. Helens, Tasmania (wild)	single	MagMAX-96 Viral RNA Isolation Kit

DNA was extracted from each sample using the corresponding kit shown in Table 1. After DNA extraction, the presence of OsHV-1 was confirmed through testing with two real-time PCR assays (Jenkins et al., 2013; Martenot et al., 2010) and a conventional PCR (Arzul et al., 2001a).

### 2.2 Short read whole genome sequencing

Extracted nucleic acid was quantified with a Qubit 2.0 fluorometer using a dsDNA high sensitivity kit (Thermo Fisher) prior to being prepared for sequencing using a Nextera XT DNA library preparation kit, following the manufacturer’s recommendations. DNA concentrations and fragment sizes of the prepared library were measured using an Agilent Bioanalyzer chip and a Qubit High Sensitivity dsDNA kit, and pools were diluted to 2 nM prior to sequencing.

All pools collected before 2024 were loaded onto a NextSeq 1000/2000 P1 600-cycle cartridge and sequenced on a NextSeq 2000. All pools collected in 2024 were loaded onto a MiSeq V2 300-cycle standard kit.

### 2.3 Long read amplicon sequencing

To resolve repeat boundaries, regions of low similarity to the OsHV-1 RefSeq genome, and a putative large deletion in the short-read assemblies, we amplified these targeted regions using long-range PCR with custom primers and sequenced the amplicons using Nanopore long reads. Based on the draft genome generated from short read sequencing for OSHV_NSW_2013_1, eleven primer pairs were designedusing the Geneious Prime function ‘design new primers’, which uses Primer 3 (Untergasser et al., 2012). The user provided optimal settings were with the optimal settings of T_m_ 56–61°C, minimum hairpin T_m_, and self-dimer T_m_. Six primer sets were designed to cross different repeat regions within the draft genome (e.g., TR_L_, IR_L_ IR_S_,TR_S_) to confirm the start and end of each region (Supplementary materials I). Second, two primer sets were designed to cover two regions where the regions showed low localised pairwise similarity with the OsHV-1 RefSeq genome AY509253.2 (Davison et al., 2005) (Supplementary materials I). Third, a large deletion of ∼ 5000 bp was observed in the draft genomes assembled which was not present in the OsHV-1 RefSeq genome AY509253.2. Three primer pairs were designed to investigate this region: one mapping across at either ∼ 6000 bp or 1000 bp, depending on the true presence of the deletion, and two sets designed to amplify the deleted region if it was present (Supplementary material I). The predicted primer product size varied for each pair but ranged from 1612 bp (OsHV-1_primer_pair_1) to 5180 bp (OsHV-1_primer_pair_7) (Supplementary material I). All primer sets designed are mapped and shown in Supplementary material II. PCR amplification was attempted for all primer pairs in three samples selected based on their relatively high DNA concentrations, high proportions of viral reads relative to non-viral reads, and suitable Bioanalyzer fragment-size profiles: OsHV_TAS_2016_1, OsHV_TAS_2019_1 and OsHV_TAS_2019_2.

PCRs were performed using HotStarTaq Master Mix Kit (QIAGEN) under the following conditions: 95°C for 15 min, 35 cycles of 94°C°C for 30sec; 55°C for 30sec; 68°C for 2 min, and 68°C for 7 min. Nine primer pair products were successfully amplified, but the two primer sets designed to amplify parts of the 5000 bp region found in AY509253.2 failed. Amplified PCR products were quantified on a Qubit 2.0 fluorometer using a Broad Range Sensitivity kit and pooled into two pools per sample. Each pool was then prepared for long read sequencing using a Native Barcoding Kit 24 V14 (SQK-NBD114–24) and run on a R10.4.1 MinION flow cell using a Mk1B MinION device following protocol version “NBE_9169_v114_revO_15Sep2022 for the Ligation sequencing gDNA - Native Barcoding Kit”. The MinION sequencer was run for two hours producing a minimum read depth of 1000 reads per amplicon, per sample before stopping.

### 2.4 Collection of New Zealand raw sequence data

The raw FASTQ data of three OsHV-1 genomes from New Zealand was publicly available on the NCBI Sequence Read Archive under the Accessions SRR14210303, SRR14210304, and SRR14210305 (Table 2). This data was previously used to generate mutation rates of OsHV-1 but not assembled into complete genomes (Morga et al., 2021). The raw FASTQ data was sequenced as 100 bp paired-end reads on an Illumina HiSeq machine (Morga et al., 2021). This data was downloaded and analysed using the same method as the NextSeq and MiSeq data generated in this study.

**Table 2:** Summary table of Australian and New Zealand OsHV-1 genomes sequenced including pre genome assembly and post genome assembly statistics. Pregenome assembly statistics are as follows, preliminary Qubit concentrations pre library preparation, host to viral reads sequenced ratios, number of contigs > 1 kbp generated through SPAdes, number of contigs > 1 kbp assigned to an Oyster species (family Ostereidae) through BLASTx, number of contigs >1 kbp assigned as OsHV-1 through BLASTx. Post genome assembly statistics are as follows; genome length, GC nucleotide content of genomes, GC content of all reads used to construct each genome, total reads aligned, read depth and % of missing bases based on a read depth minimum cut-off of 5. *Given the high estimated missing data rate these genomes are considered partial genomes and the genome length is an estimate which may vary slightly from that reported.

Sample	Qubit concentration pre library preparation (ng/µl)	Ratio of host reads to viral reads sequenced	Contigs >1 kbp generated	Contigs >1 kbp generated assigned to Ostreidae family member	Contigs >1 kbp assigned as OsHV-1 by BLASTx	Genome length	GC content genome %	Total reads aligned	sequencing depth median	% genome bases at ≥1 read depth	% genome bases at ≥5 read depth
**OsHV_NSW_2010_1**	0.896	7879.8:1	340	298	36	202,752*	38.87	15,077	15	94.07	86.51
**OsHV_NSW_2011_1**	1.89	64.8:1	155	151	3	202,760	38.57	266,805	152	100	99.57
**OsHV_NSW_2013_1**	3.37	18.1:1	37	34	2	202,795	38.60	2148,721	2673	100	99.99
**OsHV_SA_2018_1**	0.82	212.6:1	53	49	4	202,758	38.59	382,851	319	100	99.98
**OsHV_TAS_2016_1**	4.77	3.6:1	108	101	6	202,769	38.58	3692,747	2742	100	100
**OsHV_TAS_2016_2**	0.765	98.5:1	25	19	4	202,770	38.59	241,697	124	100	99.70
**OsHV_TAS_2018_1**	0.214	64.4:1	85	80	4	202,758	38.58	72,012	48	100	99.20
**OsHV_TAS_2019_1**	0.86	212.6:1	72	51	15	202,764	38.59	102,948	41	100	98.20
**OsHV_TAS_2019_2**	0.298	1.1:1	85	64	5	202,771	38.60	4655,627	2575	100	100
**OsHV_TAS_2024_1**	1.84	33.7:1	198	95	90	202,848*	38.60	21,490	12	96.57	92.53
**OsHV_TAS_2024_2**	2.13	26.4:1	194	156	36	202,771*	38.67	19,454	12	99.03	91.74
**OsHV_TAS_2024_3**	1.51	436:1	229	92	110	202,842*	38.88	15,413	5	86.74	54.58
**SRR14210303**	NA	139.6:1	202	187	5	202,885	38.58	192,003	94	100	99.99
**SRR14210304**	NA	31.2:1	11,753	11,059	6	202,883	38.58	292,320	145	100	100
**SRR14210305**	NA	233.4:1	176	170	3	202,877	38.58	123,727	61	100	99.98

### 2.5 Data filtering and de novo assembly of short-read data

Raw FASTQ reads were filtered and their adaptors trimmed using fastp *v0.23.2* (Chen et al., 2018) with the following settings: minimum length > 75, individual base minimum quality filter of Q15, and a front sliding window of four bases with an average minimum quality filter of Q20. Post filtering, reads were aligned against the Pacific oyster genome (GCA_025765685.3, (Qi et al., 2023)) to filter out host reads using Bowtie 2 *v2.4.5* (Langmead and Salzberg, 2012) with default settings and very sensitive mode. The non-aligned reads were then assembled into contigs using SPAdes *v3.15.5* using default settings and “–careful” option (Bankevich et al., 2012). All assembles were then searched against the NCBI non-redundant protein database (June 2024 release) using DIAMOND *v2.0.15* blastx under the preset sensitive settings (Buchfink et al., 2015) to identify the closest matching OsHV-1 whole genomes to assembled contigs. The assembled contigs were then aligned directly to the following OsHV-1 genomes in Geneious Prime *v2020.2.5* using the MAFFT plugin with open gap penalties of 2 and extension penalties of 0.12 to investigate potential gaps within the assembly: (AY509253.2 [current RefSeq genome of OsHV-1], MF509813.1 [The NCBI reference which matched with highest similarity to references during DIAMOND blastx).

Following alignment, the IR_L_/TR_L_ (internal and terminal repeats of the long region) and IR_S_/TR_S_ (internal and terminal repeats of the short region) were each assembled as a single copy, as expected for highly similar duplicated regions. An inverted copy of each assembled repeat was provisionally placed at the corresponding position in the complete genome representation. Because the major repeat units exceeded the span of the paired-end Illumina reads, their positions and orientations were not considered resolved from the short-read assemblies alone and were subsequently evaluated using targeted long-read amplicons spanning the repeat-to-unique-region junctions, as described in Sections 2.3 and 2.6. The paired-end 300-bp Illumina reads were used to reconstruct the shared repeat sequence and assess local nucleotide support, while BBMap results showed perfect ambiguous dual mapping across corresponding repeat copies. No copy-specific sequence differences were inferred from ambiguously mapping reads.

Completed genomes were indexed using Bowtie 2, and the quality-filtered Illumina reads were aligned back to their corresponding genomes using Bowtie 2 *v2.4.5* with the settings described above. An initial consensus was generated using the consensus function in SAMtools v1.16.1 (Li et al., 2009) in simple mode, with a minimum read depth of 1 and a call fraction of 50%. This initial low-depth consensus was used to recover the basic genome structure, minimise unresolved positions that could interfere with subsequent read alignment, and facilitate inspection of the assembled regions. The reads were then realigned to this intermediate consensus, and the resulting alignment files were imported into Geneious Prime to assess local read support across the provisionally placed IR_L_/TR_L_ and IR_S_/TR_S_ regions and their junctions. Specifically, the alignments were examined for abrupt termination of read support at repeat-region boundaries, which would indicate an unsupported join. No abrupt termination of short-read support was observed at the repeat-region boundaries; however, structural validation of the repeat-to-unique-region junctions was further supported based on targeted long-read data described below (Section 2.6).

Following this inspection, the reads were remapped to each revised consensus genome, and the final consensus sequences were generated using SAMtools consensus in simple mode, with a minimum read depth of 5 and a call fraction of 66%. Consequently, positions represented in the final genomes were required to have a minimum read depth of 5, while positions below this threshold were reported as ambiguous bases rather than being called from the initial minimum-depth consensus. Inspection of the initial and final assemblies indicated that the refinement predominantly involved minor adjustments to a small number of homopolymer tract lengths per genome rather than substantial changes to genome structure.

Accordingly, iterative remapping of the quality-filtered Illumina reads, inspection of the resulting alignments, and consensus updating were used to refine the draft assemblies and ensure that the final sequences were supported by the originating read data, consistent with the refinement of Illumina-derived herpesvirus assemblies described by Li et al. (2023). Targeted inspection and long-read validation of the IR_L_/TR_L_ and IR_S_/TR_S_ repeat regions were performed separately because repeat-associated junctions are recognised assembly challenges in complex herpesvirus genomes (Nanamiya et al., 2024; Rajagopalan et al., 2022).

### 2.6 Data filtering and mapping of long-read data

Raw FAST5 reads basecalled to FASTQ and adapter-trimmed using Guppy-basecaller *v6.5.7* (Wick et al., 2019) and quality filtered using a cut-off quality score of Q12, minimum length of 800 bp, and maximum length of 5000 bp using NanoFilt *v2.8.0* (De Coster et al., 2018). Filtered FASTQ reads were then aligned to the generated consensus genomes using Geneious Prime reference mapping and inspected manually to edit/confirm the overall structure of each OsHV-1 genome around each join between major repeat regions. Long-read mapping was consistent with the assembled genomes, and so no structural changes were made as a result of long read data.

### 2.7 Generation of summary statistics for each genome

All raw short and long reads were mapped against each consensus genome using BBTools *v39.01* (Bushnell, 2014) function BBMap with metrics for median coverage, and percentage of genome passing with read depth ≥ 1 and read depth ≥ 5 reported using the BBTools script pileup.sh. This statistic was derived using the final assembled genomes generated after a SAMtools consensus of read depth 1 and call rate 50%. This was done so that the minimum number of ambiguous sequences were present prior to estimating coverage so that tracks of Ns did not cause reads to fail to align when they overlapped with these regions. The final reported genomes are the final polished genomes with the read depth filter of 5 with statistics generated from aligning to the most complete genomes possible for each sample (no read depth filter causing poor alignments around each ambiguous N string). For genomes where complete genome coverage was not achieved, the other genomes in this study were used as a guide for the size of the N region not covered. For these regions, a manual check of read pairs performed to investigate whether read pairs were mapping across the generated N region (indicative of a true deletion), and if there was no evidence of true deletion, the N region was kept. Coverage plots for each assembled Australian genome are present in Supplementary material III.

### 2.8 Iterative genome refinement and whole-genome phylogenetic analysis

Consensus genomes and all available OsHV-1 whole genomes (present as of January 2024) downloaded from NCBI (see Supplementary materials IV) were aligned using MAFFT *v7.508* (Katoh et al., 2002) with an open and extension penalty of 2 and 0.12 respectively, up to 1000 iterations, and 150 ‘retree’ calibrations. A phylogenetic tree of the alignment was then generated using IQ-TREE *v2.2.0.3* (Nguyen et al., 2015) with 2000 bootstraps, 2000 SH-aLRT tests and using a substitution model identified automatically through ModelFinder in IQ-TREE (Kalyaanamoorthy et al., 2017). Trees were then visualised using the R packages ggtree *v3.7.1.00* in R *v4.2.2* (R Core Team, 2023; Yu, 2020).

### 2.9 Investigation of putative recombination events in assembled genomes

Potential changes in phylogenetic signal across the whole-genome alignment were investigated using GARD in HyPhy v2.5.100 (Kosakovsky Pond et al., 2006). GARD is a likelihood-based model-selection method that evaluates whether an alignment is better explained by a single phylogenetic history or by multiple partitions separated by putative breakpoint positions. Breakpoints identified by GARD were therefore treated as candidate changes in phylogenetic signal rather than as independently confirmed recombination events.

Because extensive missing sequence can affect breakpoint inference, OsHV_NSW_2010_1, OsHV_TAS_2024_1, and OsHV_TAS_2024_3, which contained the three highest proportions of missing data among the study samples, were excluded from the GARD analysis. OsHV_TAS_2024_2 also contained a relatively high proportion of missing data but was retained so that at least one genome from each sampling location and collection period remained represented. The remaining genomes were aligned using MAFFT with the settings described above, and GARD was run in the preset “Faster” mode with a maximum of 20 allowable breakpoints.

A maximum-likelihood phylogeny was inferred from the complete GARD input alignment using IQ-TREE with the settings described above and was used as the baseline tree. The preferred breakpoint model was selected by GARD using the improvement in Akaike information criterion. The resulting breakpoint coordinates were used to define alignment partitions, and a separate maximum-likelihood tree was generated for each evaluable partition using IQ-TREE, with the nucleotide substitution model selected automatically by ModelFinder and 1000 ultrafast bootstrap replicates.

Partition-specific trees were compared with the baseline tree after both trees were pruned to an identical set of taxa. In addition to inspection of the major phylogenetic groupings, tree congruence was quantified using four complementary measures: normalised Robinson-Foulds distance to assess differences in internal splits, Spearman correlation of pairwise patristic distances to assess preservation of relative evolutionary distances, scaled branch-length normalised root-mean-square error to quantify branch-length differences after accounting for variation in overall substitution rate, and quartet concordance to assess agreement among resolved four-taxon relationships. Internal-split comparisons were additionally repeated at node-support thresholds of 50%, 80%, and 90%, with supported-split precision and recall used to distinguish strongly supported topological conflict from reduced phylogenetic resolution within shorter partitions. GARD partitions were not interpreted as confirmed recombinant regions because changes in phylogenetic signal may also arise from localized sequence divergence, structural variation, repetitive sequence, alignment uncertainty, or insufficient phylogenetic information. The purpose of this analysis was to determine whether localized variation in phylogenetic signal materially affected the principal whole-genome relationships, rather than to reconstruct individual recombination events or identify parental and recombinant lineages. Phylograms of every genome split region and quantitative statistics of concordance of phylogenetic signal are available in Supplementary material V.

### 2.10 Genome annotation and core gene analysis

Complete OsHV-1 genome assemblies were annotated using a multi-stage, reference-guided and homology-assisted workflow rather than a single-reference similarity transfer step. For each sample, annotations were first generated independently against three OsHV-1 reference genomes (MF509813.1 [chosen as it is the most closely matching reference by pairwise identity], AY509253.2 [chosen as it is the NCBI RefSeq reference and first OsHV-1 whole genome sequence], and KY242785.1 [chosen as an example sequence of known OsHV-1 *‘*μVar’) using the annotation transfer program Liftoff *v1.6.3* under default settings (Shumate and Salzberg, 2021).

Per-reference annotation outputs were then post-processed using custom Python workflows to improve boundary accuracy and recover genes missed or incompletely transferred by direct liftover. First, a truncate-aware correction step was applied to each per-reference GFF3 file to identify CDS interrupted by terminal ambiguous sequence stretches or internal premature stop codons. In these cases, CDS boundaries were trimmed to the supported coding region, and partial coding sequences were flagged where the 5′ or 3′ end overlapped unresolved assembly sequence. This step was implemented in a custom python script which was developed specifically for these OsHV-1 assemblies.

Second, annotations likely to be artefacts of assembly gaps near the start codon were excluded from the primary per-reference annotation set and subjected to a separate partial-gene rescue procedure. In this rescue step, protein homology searches were used to identify partial but biologically plausible coding regions even when complete start or stop codons were absent from the assembly. Candidate partial genes were screened against the genome using tblastn from the NCBI BLAST**+** *v2.17.0* (Madden, 2013), and retained when they matched a substantial (75%) portion of the expected protein-coding region from any OsHV-1 genome returned by tblastn. Rescued loci were then re-annotated as partial CDS, with the missing 5′ or 3′ boundary explicitly recorded in the annotation.

After per-reference correction and gap-aware rescue, the three annotation sets were compared and merged. The MF509813.1-derived annotation was used as the base annotation set, and genes absent from that set but confidently detected in the corresponding sample using the AY509253.2 or KY242785.1 references were added in genomic order using a custom merge workflow in Python. This merge step was restricted to genuinely additional loci and was designed to avoid replacing already annotated truncated loci with inappropriate full-length donor models from alternative references. For genes present in all three reference-derived annotation sets, differences in the transferred start or stop coordinates were flagged for manual inspection. Candidate boundaries were compared against the assembled genome sequence, including the positions of in-frame start and stop codons and the corresponding reference-derived alignments. In all cases, the conflicting boundaries could be resolved by identifying the candidate boundary that was consistent with the coding sequence of the assembled genome. This step served as a secondary validation of the automated annotation boundaries rather than as a separate annotation procedure.

Because some loci still required case-by-case correction or validation after automated processing, a final manual curation step was applied to the merged annotations. This final stage was performed where one or more of three conditions were met: (1) annotation boundaries differed among the three reference-derived annotation sets; (2) the ORF was >30% shorter than the corresponding reference gene, or; (3) ambiguous sequences were present within 10 bp upstream or downstream of the predicted start or stop codon. In this stage, selected genes were inspected manually and all possible start/stop codon combinations were extracted and run through BLAST+ tblastn and the start/stop codon combination with the highest alignment was kept, with boundaries adjusted accordingly. Finally, novel ORFs not recovered by reference-guided annotation were identified using Prodigal *v2.6.3* under default settings (Hyatt et al., 2010).

For the NCBI reference genomes, candidate homologous CDSs were identified and extracted using the annotation names associated with the corresponding public records; these genomes were not processed through the complete annotation workflow applied to the newly assembled genomes. Each extracted reference CDS was manually inspected to confirm that it represented the expected gene, was complete, and was suitable for like-for-like comparison with the corresponding CDSs from the other genomes. A subset of genes was then retained from all samples and reference genomes under the following conditions: (1) the gene was present in >90% of genomes; (2) the gene was completely sequenced, with no ambiguous bases present within the CDS, in >75% of the Australian and New Zealand genomes analysed here, and; (3) the gene was excluded if premature stop codons associated with homopolymer regions were observed in multiple Australian or New Zealand samples (e.g., a homopolymer site appears to have fewer or more bases in one sample genome and this site results in a frameshift and premature termination of a CDS). The third condition was applied because homopolymer regions have elevated sequencing and assembly error rates, making premature stop codons in these regions more likely to represent technical artefacts. Genes showing substantial length differences among samples were also excluded where these differences prevented a like-for-like comparison of homologous sequence. Lastly, for CDSs with multiple copies due to being present in a repeat region, only one member of each was retained. All retained genes were aligned individually using MAFFT v7.508 with the settings described above.

The individual gene alignments were then concatenated, and a combined core-gene phylogeny was generated using IQ-TREE with a partition file delineating the start and end of each gene alignment and allowing an independent nucleotide substitution model to be selected for each partition. The resulting partitioned maximum-likelihood tree was visualised using ggtree. This gene subset was not intended to represent every gene conserved across > 90% of the genomes. Instead, it represents a more stringently filtered subset of conserved genes that were also sufficiently complete and comparable across the Australian and New Zealand genomes, thereby reducing biases associated with low sequencing depth, ambiguous bases and comparisons between substantially different gene sequences. Existing annotations from the public genomes were therefore used to identify candidate homologous CDSs, while final inclusion was based on manual assessment of gene identity and completeness, followed by direct comparison within the individual CDS alignments. A complete list of genes and their status as retained or excluded from the core-gene dataset is provided in Supplementary Material VI.

To identify whether the phylogenetic signal of the whole-genome and core-gene analyses were impacted by the coverage of lower coverage assembled genomes, a secondary sensitivity test utilising only complete genes at a coverage of ≥ 10 across all study assembled genomes and across the entire gene was performed. This analysis was performed using the settings for alignment and phylogenetic signal described above in 2.9 (Supplementary material VII).

### 2.11 OsHV-1 marker gene comparison

Two marker-region datasets were generated to address the differing availability of historical OsHV-1 sequence data and to evaluate how well commonly used classification markers reflected the whole-genome phylogeny. The C-region-only dataset was used to maximise sample number and geographic representation because this region has historically been sequenced more widely than other OsHV-1 genomic regions. The five-region dataset contained fewer samples but provided substantially more sequence information and allowed the genomes generated here to be compared directly with previously characterised Australian genotypes which sequenced samples for these added 5 genomic regions. The two datasets therefore provided complementary comparisons of marker performance, historical sample diversity and existing Australian OsHV-1 diversity.

Each of the five regions commonly used for OsHV-1 classification were extracted separately (C-region, A-region [the region between ORF42–43], non-coding region between ORFs49–50, ORF35–38, and ORF88) to make two distinct datasets for additional alignment and phylogenetic tree generation: one C-region exclusive tree and one 5 region tree (Bai et al., 2016; Martenot et al., 2013, 2012; Trancart et al., 2023). For the C-region analysis, sequences were downloaded from NCBI Virus using the search terms “C-region” or “ORF4” and filtered using a minimum length threshold of 400 bp, resulting in 57 targeted C-region accessions. An additional five C-region sequences were identified through a literature search of studies investigating OsHV-1 genetic diversity. The C-region was also extracted from the 15 genomes assembled in this study and from 30 publicly available OsHV-1 whole genomes, initially producing 107 records. Of these, 26 represented the same sample twice because NCBI had assigned separate nucleotide accessions to C-region annotations associated with corresponding whole-genome records. These duplicate sample representations were identified before alignment and removed so that each sample was represented only once, resulting in a final dataset of 81 sequences. The sequences were aligned using MAFFT with the settings described above and trimmed to common C-region boundaries in Geneious Prime so that all retained sequences covered the same homologous region. The resulting alignment was used to generate a maximum-likelihood tree using IQ-TREE with the settings described above and was visualised using ggtree (see Supplementary materials IV for list of all accessions downloaded). For the five-region dataset, all completed genotypes from a recent Australian study (Trancart et al., 2023) were downloaded and aligned with the extracted regions from all whole genomes. For each region, a separate MAFFT alignment was performed (with settings described as above). All genes were then concatenated and a multi-region phylogenetic tree was created using independent substitution models per gene using IQ-TREE with a partition file delineating the start and finish of each separate tree. A final tree showing a partitioned maximum-likelihood tree of all individual regions was then plotted in ggtree. The corresponding genomic coordinates for each trimmed region are given according to the OsHV-1 RefSeq genome AY509253.2 and are C-region 4195–4713 bp, A-region 60,001–60,508 bp, non-coding region between ORFs49–50 72,467–72,870 bp, ORF35–38 52,031–52,906 bp, and ORF88 133,242–134,062 bp.

### 2.12 Pairwise identity comparisons

Pairwise identity profiles were generated to identify and quantify the genomic regions contributing to the differences observed among the major whole-genome phylogenetic groups. This analysis allowed for the visualisation of whether divergence between the Australian/New Zealand genomes and representative OsHV-1 genomes was distributed uniformly or concentrated within particular genomic regions, including regions containing large insertions or deletions.

Based on the results of the whole genome analysis, four NCBI reference genomes were chosen to represent key branches of defined phylogenetic structure, MF509813.1, (China 2001) MW412420.1 (China 2019), AY509253.2 (France 2003), and OM811597.1. Pairwise identities between each sample and the NCBI reference genomes chosen were calculated using a custom python script using Biopython. All genomes were split into 2000-bp chunks and the average pairwise identity of each chunk was reported. Pairwise identity was calculated as 100 × (identical positions) / (aligned positions + internal gap positions) and with ambiguous bases (Ns) being ignored (not contributing as either identical or non-identical and being subtracted from the total length).

## 3 Results

### 3.1 Summary of genomes assembled

Fifteen Australian and New Zealand OsHV-1 genomes were constructed, comprising 11 complete genomes and four partial genomes. Genomes with less than 95% of their sequence supported at a minimum read depth of 5 were classified as partial. On this basis, OsHV_NSW_2010_1, OsHV_TAS_2024_1, OsHV_TAS_2024_2, and OsHV_TAS_2024_3 were classified as partial, with 86.51%, 92.53%, 91.74%, and 54.58% of their respective genomes supported at a read depth of ≥5. The remaining 11 genomes were classified as complete. Median sequencing depth varied from 5 for OsHV_TAS_2024_3 to 2742 for OsHV_TAS_2016_1, while the proportion of missing bases ranged from 0% in four samples to 9.53% in OsHV_TAS_2024_3. Genome length differed by a maximum of 133 bp between SRR14210303 and OsHV_NSW_2010_1, representing approximately 0.06% of the average genome length. However, because the four partial genomes contained unresolved sequence, their reported lengths should be considered estimates (Table 2). A total of 113 genes, excluding fragmented genes and genes that did not meet the completeness criteria, were retained for the core-gene phylogeny and collectively comprised approximately 71% of the complete genome length.

### 3.2 Phylogenetic relationships between Australian, New Zealand and other OsHV-1 genomes

GARD identified nine putative breakpoint positions across the OsHV-1 whole-genome alignment, dividing the alignment into ten partitions ranging from approximately 1600 to 89,000 bp in length. These putative breakpoints represent positions at which allowing separate phylogenetic histories improved model fit and were not interpreted as nine independently confirmed recombination events.

Partition 8, an approximately 2000-bp region, was not used for phylogenetic reconstruction because only MW412420.1 retained sequence across the partition after missing-data filtering. The region represents an insertion present in MW412420.1 but absent from the other genomes in the alignment.

For the remaining nine evaluable partitions, the principal phylogenetic groupings observed in the baseline whole-genome tree were retained. Quantitative comparisons of partition-specific and baseline trees using normalised Robinson-Foulds distance, Spearman correlation of pairwise patristic distances, scaled branch-length normalised root-mean-square error, and quartet concordance provided additional support for the general consistency of the principal phylogenetic relationships across the nine evaluable genome partitions (Supplementary Material V). Mean Spearman correlation and quartet concordance across these partitions were 0.90 and 0.94, respectively, while support-filtered split comparisons indicated that much of the remaining topological variation reflected reduced resolution or rearrangement of weakly supported nodes rather than strongly supported conflicting relationships. These results indicate that the principle whole-genome topology was not dependent on a single evaluable region of localized phylogenetic signal. However, they do not establish whether the putative breakpoint signals represent genuine recombination, localized divergence, structural variation, repetitive sequence, or alignment uncertainty.

**Fig. 2:**
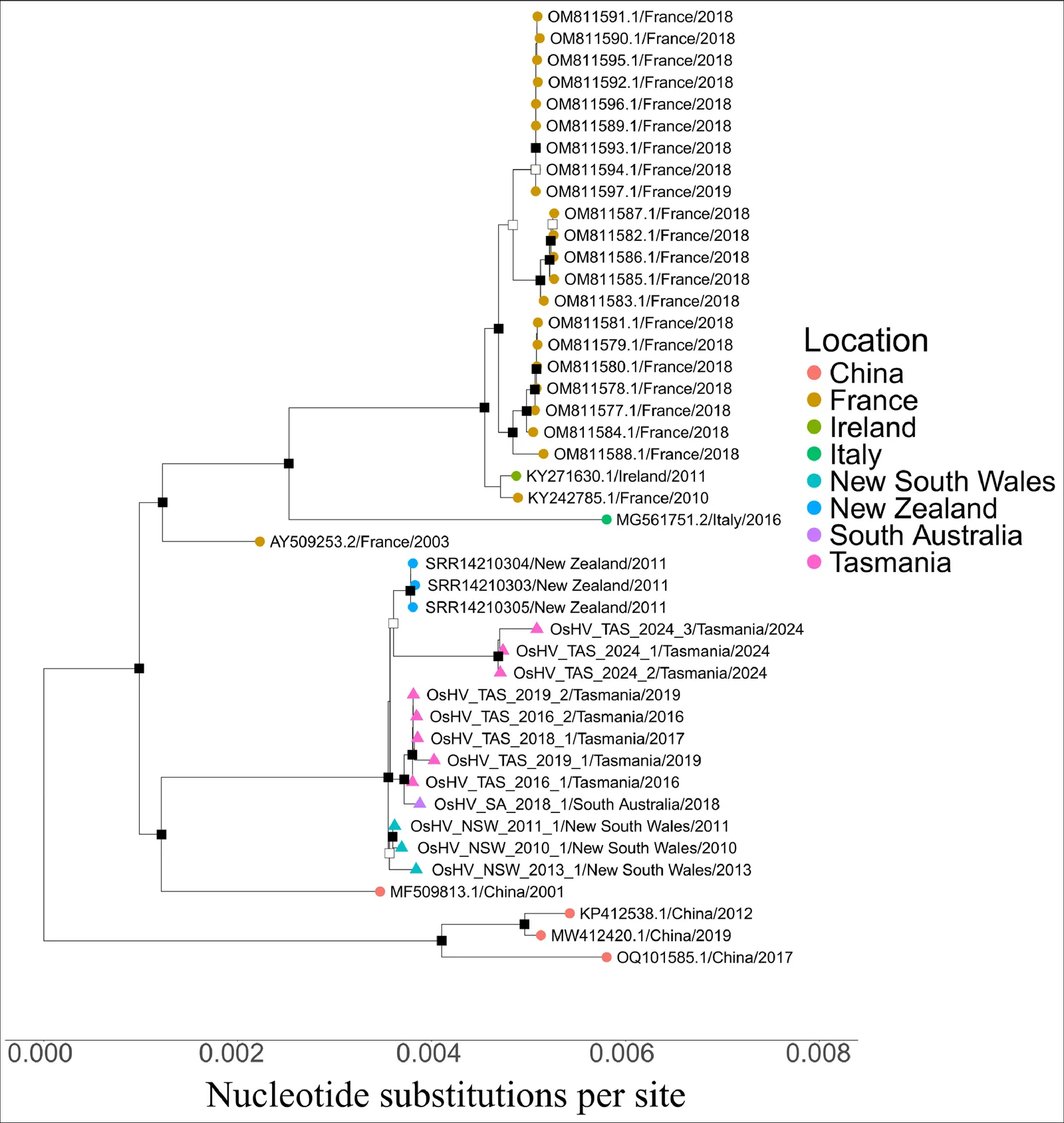
Mid-rooted whole-genome maximum likelihood phylogram of OsHV-1 genomes sequenced/assembled in this study and all available OsHV-1 whole genomes sequences available from the NCBI. The *x*-axis displays the number of substitutions per site. The phylogram was generated using the HKY+F+I substitution model. Nodes with an open square displayed > 80% confidence from SH-aLRT and ultra-fast bootstrap supports, while nodes with a closed square displayed > 95% confidence from SH-aLRT and ultra-fast bootstrap supports. Sequences generated in this study are denoted with a triangle, while NCBI reference sequences are denoted with a circle.

**Fig. 3:**
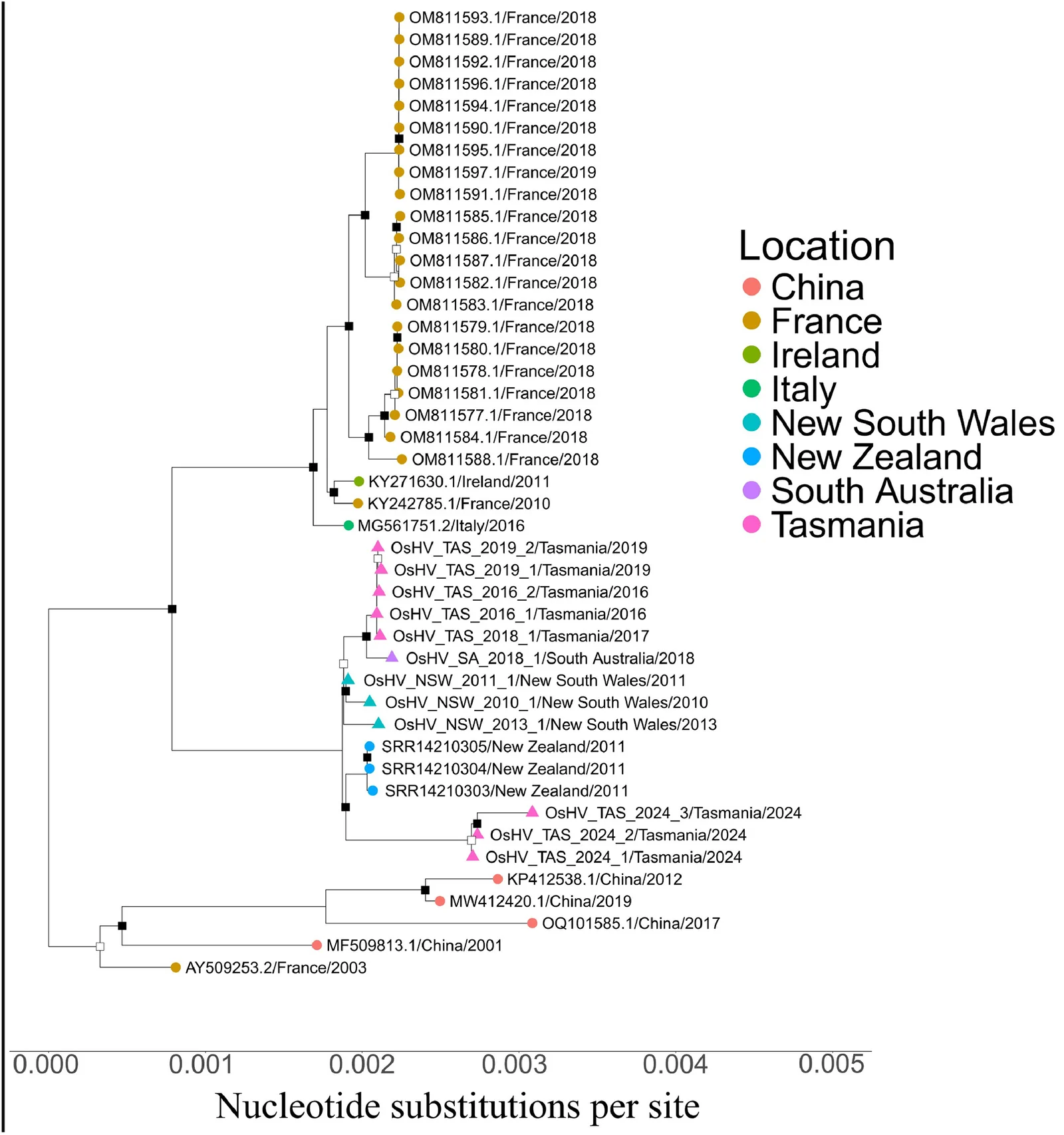
Mid-rooted core-gene genome maximum likelihood phylogram of OsHV-1 genomes sequenced/assembled in this study and all available OsHV-1 whole genomes sequences available from NCBI. The x-axis displays the number of substitutions per site. The phylogram was generated using a HKY+F+I substitution model. Nodes with an open square displayed > 80% confidence from SH-aLRT and ultra-fast bootstrap supports while nodes with a closed square displayed > 95% confidence from SH-aLRT and ultra-fast bootstrap supports. Sequences generated in this study are denoted with a triangle while NCBI reference sequences are denoted with a circle. The core gene subset generated was composed of 113 genes.

The whole-genome and core-gene analyses (113 genes) both show Australian and New Zealand sequences as a distinct clade separate to previously sequenced genomes available on the NCBI, although the exact positioning of the clade varies between trees (Fig. 2, Fig. 3, both at > 95% support for node). The whole-genome phylogeny groups most genomes from China (KP412538, MW412420, OQ101585, at > 95% support for node) as the most diverged clade from the Australian and New Zealand clade followed by a distinct clade encompassing all European genomes (> 95% support for node), and shows MF509813 as the closest neighbour phylogenetically. The core-gene phylogeny similarly clusters most Chinese sequences (KP412538, MW412420, OQ101585, > 95% support for node) as distinct, but places the Australian sequences as a separate, adjacent branch to all European sequences (excluding AY509253, > 95% support for node) (Fig. 2, Fig. 3). Within the core-gene phylogeny, both AY509253 and MF509813 were positioned as out branches before the other clades (Fig. 3, > 80% support for AY509253 and > 95% support for MF509813 node). This difference likely reflects the genomic regions retained in each analysis. The whole-genome alignment includes structural and non-coding variation, including the approximately 6.5-kbp deletion shared between MF509813 and the Australian/New Zealand genomes, whereas the core-gene analysis is restricted to conserved and sufficiently complete coding regions. The shared deletion is therefore a likely contributor to the closer placement of MF509813 in the whole-genome phylogeny, although other structural and non-coding differences may also contribute.

Within the Australian and New Zealand clades, evidence of both geographical based genetic structuring was observed between Australian states and the two countries, with sequences taken from Tasmania clustering into two different clusters representing samples collected between 2016 and 2019, and those collected after 2023 (Fig. 2, Fig. 3, all at support nodes > 80%). In the former group, the one sample from South Australia sits within a larger monophyletic clade including those Tasmanian samples. For the latter group, the three Tasmanian samples collected in 2024 are set apart from all other samples but branch off a shared basal branch with the New Zealand samples from 2011. The samples from New Zealand (2011) and New South Wales (2010–2013) each form a separate cluster which sits closest to the basal branch of the Australian/New Zealand monophyletic group. The whole-genome and core-gene phylogenies therefore recover a consistent Australian/New Zealand clade and broadly similar internal subclustering, although the placement of this clade relative to MF509813 and the European genomes differs between analyses. Both the whole-genome and core-gene phylogenetic trees reflect the same general structure; however, the nucleotide divergence of samples within the Australian/New Zealand clade are shallower in the core-gene phylogenetic tree than in the whole-genome phylogenetic tree (Fig. 2, Fig. 3). Based on the plotted substitution scales, branch lengths separating samples within this clade are generally compressed from approximately 5 × 10^−4^–1.2 × 10^−3^ substitutions per site in the whole-genome tree to approximately 1 × 10^−4^–5 × 10^−4^ substitutions per site in the core-gene tree, indicating an approximate two- to five-fold reduction in apparent nucleotide divergence when only the conserved core-gene subset is analysed. This reduced divergence is consistent with the more conservative composition of the core-gene dataset, which was restricted to conserved, sufficiently complete and directly comparable coding regions. In contrast, the whole-genome alignment also included non-coding sequences, insertions and deletions, structurally variable regions and duplicated repeat sequences, including the approximately 6.5-kbp deletion shared by MF509813.1 and the Australian/New Zealand genomes. The difference in branch lengths therefore likely reflects the combined exclusion of less constrained and structurally variable sequence. A sensitivity analysis restricted to complete CDS regions supported by a minimum read depth of 10 recovered the same principal topology and retained the three 2024 Tasmanian samples as a distinct cluster, providing additional support that their grouping was not solely attributable to low-depth nucleotide calls (Supplementary Material VII).

### 3.3 Phylogenetic relationships of OsHV-1 marker genes

**Fig. 4:**
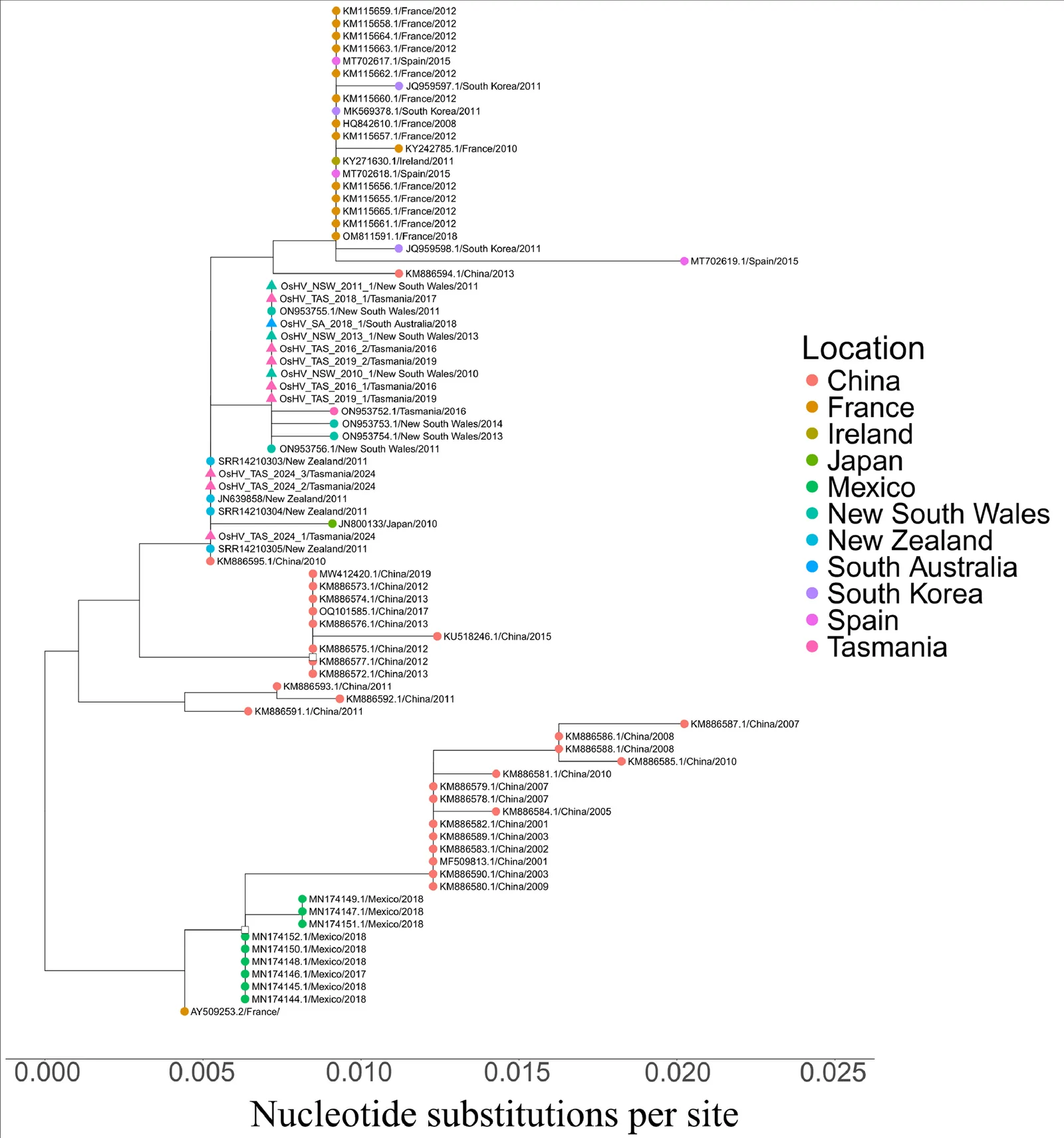
Mid-rooted maximum likelihood phylogram of OsHV-1 ORF4/C-regions sequenced/assembled in this study and all available sequences available from the NCBI. The *x*-axis displays the number of substitutions per site. The phylogram was generated using the HKY+F+I substitution model. Nodes with an open square displayed > 80% confidence from SH-aLRT and ultra-fast bootstrap supports. Sequences generated in this study are denoted with a triangle while NCBI reference sequences are denoted with a square.

**Fig. 5:**
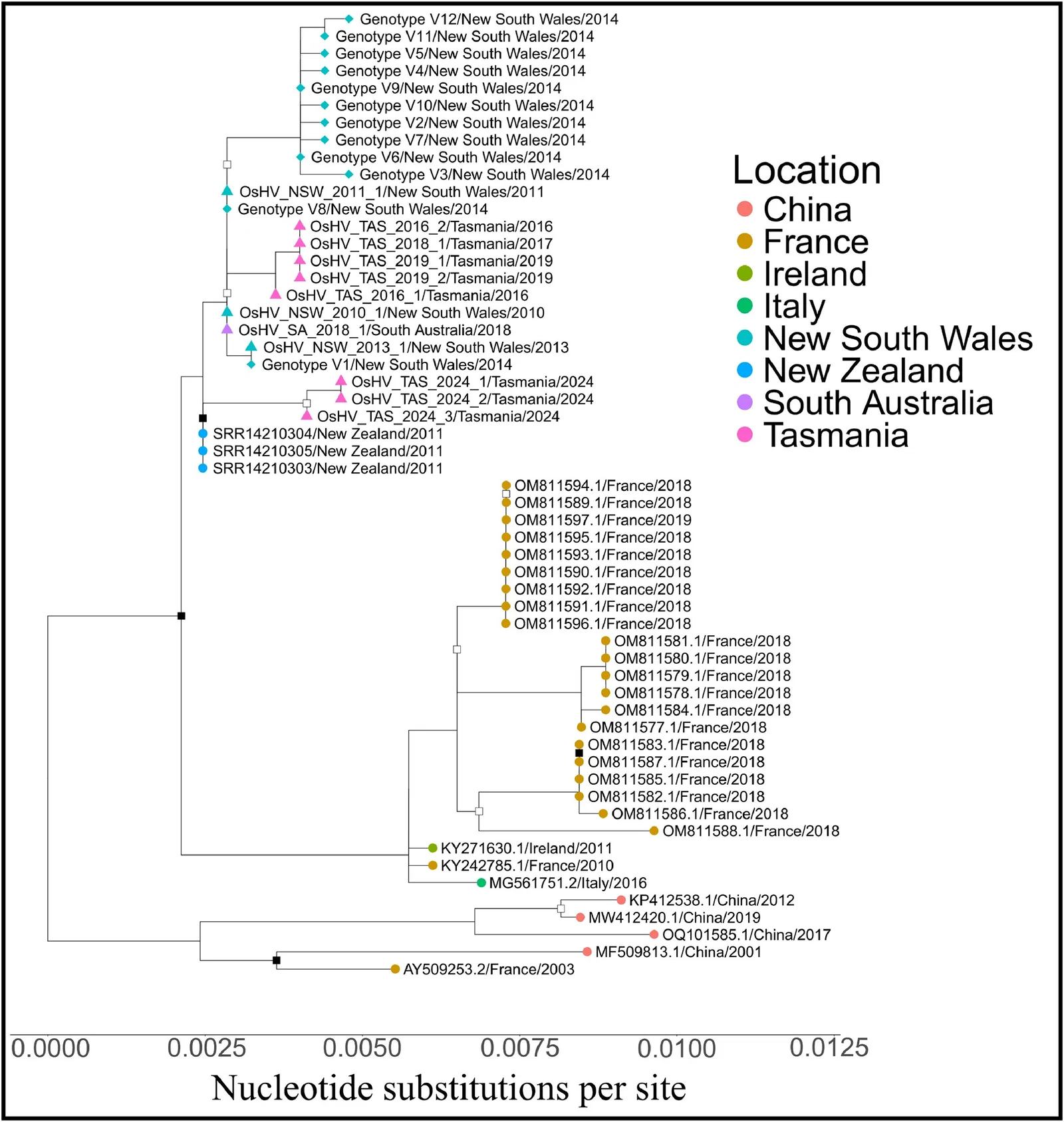
Mid-rooted maximum likelihood phylogram of five regions within the OsHV-1 genome (C-region, ORF49–50, ORF35–38, A-region, and ORF88). Samples shown include all those sequenced/assembled within this study, those which a whole genome was available on NCBI, and the 12 distinct genotypes generated from Trancart et al. (2023). The *x*-axis displays the number of substitutions per site. The phylogram was generated using a distinct substitution model per gene region. Nodes with an open square displayed > 80% confidence from SH-aLRT and ultra-fast bootstrap supports while nodes with a closed square displayed > 95% confidence from SH-aLRT and ultra-fast bootstrap supports. Sequences generated in this study are denoted with a triangle, while NCBI reference sequences are denoted with a circle, and sequences from Trancart et al. (2023) are denoted with a diamond.

Analyses of the C-region-only and five-region datasets recovered some relationships consistent with the whole-genome and core-gene analyses; however, the exact topology depended on the amount and genomic distribution of sequence analysed (Fig. 4, Fig. 5). No monophyletic group exclusively encompassing all Australian and New Zealand samples was recovered in the C-region phylogeny. Although the Australian and New Zealand samples generally clustered together, the Australian samples were placed closest to a single Chinese sample, KM886595.1, while the available Chinese samples were distributed among at least four different groups (Fig. 3). However, the node associating the Australian samples with KM886595.1 was not statistically supported, and no nodes within the C-region phylogeny received support greater than 95%. This placement was also not recovered in the five-region, core-gene or whole-genome analyses and was therefore not interpreted as evidence of a close evolutionary relationship. Instead, the poorly supported and inconsistent topology demonstrates that the C-region alone contains insufficient phylogenetic information to reliably represent the broader genome-wide relationships among these samples.

In the five-region phylogeny, which contained substantially more sequence information than the C-region analysis, the Australian and New Zealand samples formed a distinct clade that was recovered as sister to the European clade rather than to the sampled Chinese groups (Fig. 5, > 95% support for node). The difference between the C-region and five-region topologies indicates that inferred relationships are sensitive to marker selection and supports caution when classifying OsHV-1 variants or inferring broader evolutionary relationships using the C-region alone.

### 3.4 Pairwise identity comparisons

**Fig. 6:**
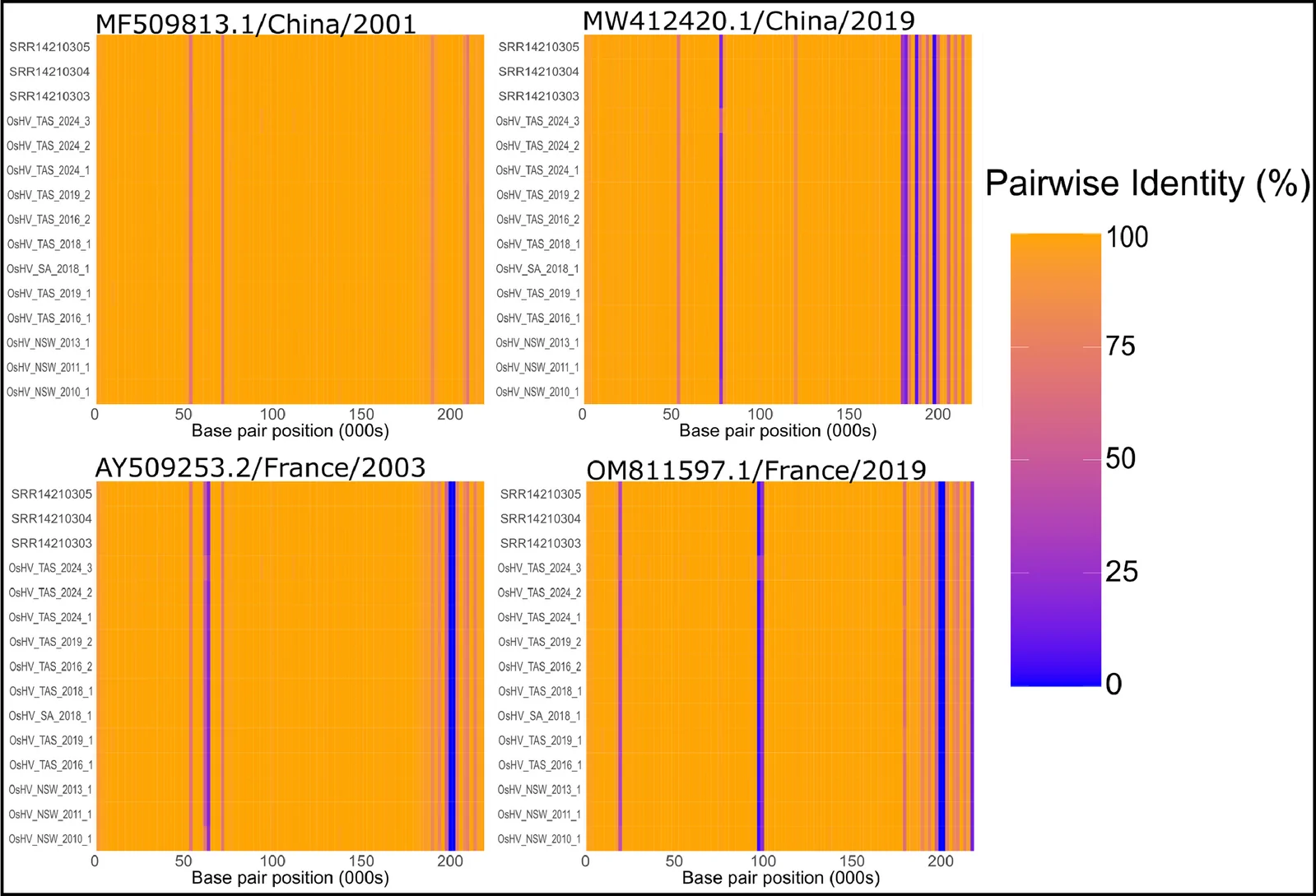
Pairwise identity of each OsHV-1 genome compared to four different NCBI reference genomes. Base-pair position in 1000s relative to the whole-genome alignment is shown on the *x*-axis. Pairwise identity was calculated as averages across 2000-bp chunks, with missing data (Ns) as neutral, and gaps in each genome being classified as dissimilarities.

The pairwise identity of sequenced OsHV-1 genomes show that over 90% of each sequenced genome from Australia/New Zealand is nearly completely conserved across each of the NCBI reference genomes (MF509813.1/China/2001, MW412420.1/China/2019, AY509253.2/France/2003, OM811597.1/France/2019) used for comparison (each marking one of the four unique clades observed in the whole-genome and core-gene phylogenetic trees). All differences between the sequenced samples and reference sequences show a similar pattern, with small to medium non-contiguous portions of the genome showing < 20% similarity, reflecting a combination of moderately sized insertions/deletions ranging from 100 bp to 5000 bp (Fig. 6). The portions of the genome showing < 20% similarity were found to be predominately located in the last ∼ 20% of the OsHV-1 genome, which coincides with part of the IR_L_ region, the IR_S_ and TR_S_ regions, the unique short region, and X region. Within this section of the genome, a ∼ 6500-bp deletion was observed for all Australian/New Zealand samples assembled here and only the reference MF509813.1 (China 2001), resulting in higher overall similarity between all samples and this reference.

The most similar reference to the sequenced genomes was found to be MF509813.1, a reference taken from a sample collected in China in 2001 (Fig. 6). Although MF509813.1 shows the highest average pairwise identity with sequenced samples, specific regions within other references were found to align more closely to sequenced samples. For example, 25–90 kbp in OM811597.1 show higher pairwise identity to the sequenced Australian and New Zealand genomes when compared to MF509813.1 (Fig. 6).

### 3.5 Novel identified ORFs and observed deviations from existing classified ORFs

Based on the ORFs (open reading frames) present in the references AY509253.2 (France 2003) and KY242785.1 (France 2010 OsHV-1 µvarA), there are between 124 and 130 putative genes within the OsHV-1 genome. A total of 123 ORFs were found in each of the fully sequenced genomes with evidence of all 123 present in each of the partially sequenced genomes, of these ORFs, 121 (out of 124 present in AY509253.2) were shared with AY509253.2, and 123 (out of 127 present in KY242785.1) ORFs were shared with KY242785.1. Comparison of each of the sequenced genomes revealed three ORFs found within either reference genome were not present in the Australian and New Zealand genomes: ORFs 36, 48 and 123. These absence patterns were not unique to the Australian and New Zealand genomes. ORF36 and ORF48 are also absent from several established OsHV-1 reference genomes, including KY242785.1, while ORF123 is absent from MF509813.1 as part of the same approximately 6.5-kbp deletion identified in the genomes assembled here. The fragmented and absent ORF patterns are therefore not interpreted as novel pseudogenisation or gene-loss events specific to the Australian/New Zealand lineage. Further, the ORFs 70, 115, 117, and 119 were found to be shortened through either frameshift or nonsense mutations. One mutation within ORF38 was found to shorten the gene by 604 bp; this mutation was present in all Australian and New Zealand samples and all references except MF509813.1 (China 2001) and AY509253.2. One large deletion of approximately 6500 bp is present within all samples assembled here and only in the reference genome MF509813.1. This deletion results in the deletion of ORFs 120–123 immediately after the IR_S_ region near the end of each OsHV-1 genome. As ORFs 120–122 are replicated before the TR_S_ region, only one ORF from this 6500-bp chunk is lost in the sequenced genomes. All ORFs affected by large predicted coding-sequence disruptions, including premature termination codons, large frameshifts or complete gene loss, had no known functional annotation. In all Australian and New Zealand genomes, including the complete genomes, ORF5, ORF32, ORF33, ORF62, ORF63 and ORF65 were consistently represented as multiple predicted coding fragments. These patterns were not associated solely with ambiguous sequence or assembly gaps and were retained following manual review of the corresponding genomic sequence and protein homology. Fragmented predicted genes have also been described in the RefSeq genome AY509253.2, indicating that this feature is not unique to the Australian and New Zealand genomes (Davison et al., 2005). These loci are therefore reported descriptively as fragmented ORFs rather than interpreted as newly pseudogenised genes. No novel ORFs were identified exclusively within any of the Australian or New Zealand OsHV-1 genomes.

Twenty-seven coding-sequence variants were identified in eight genes with known or predicted functions in the Australian and New Zealand OsHV-1 samples. Mutations were observed in eight different genes: the DNA polymerase (ORF100), RR1 (ORF51), RR2 (ORF20), exonuclease (ORF95), two IAP proteins (ORFs 42, 87), one RING finger protein (ORF9), and envelope membrane protein (ORF41) (Supplementary materials VIII). Of all the above-described mutations, five were observed exclusively within Tasmanian samples from 2024, three were observed within Tasmanian and South Australian samples from 2016 to 2019, and all others were found in all samples from Australia and New Zealand.

## 4 Discussion

Here, fifteen OsHV-1 genomes were characterised from samples collected during mortality events in Australia and New Zealand, twelve of which were newly sequenced and three of which utilised raw sequencing data from a prior OsHV-1 study (Morga et al., 2021). Although all Australian and New Zealand OsHV-1 genomes were assumed to be similar to the OsHV-1 µVar attributed to the France 2008 outbreak (Jenkins et al., 2013), whole-genome sequence and phylogenetic analyses reveal the evolution and geographic distribution of the virus is likely more complex than simply a direct spread of the same variant of OsHV-1. Instead, moderately sized indels in the OsHV-1 genome delineate regional genetic structuring, while individual SNP mutations highlight clear temporal differentiation between genomes sequenced over the past 14 years within Australia. These patterns represent associations between genomic variation, sampling location and collection period but do not independently establish the direction or timing of viral movement. Although several large predicted ORF-disrupting mutations were observed, very few genes in OsHV-1 are functionally annotated, and no large predicted ORF-disrupting mutations (i.e. large frame shifts/deletions/insertions) were observed in any gene with a known function. When considering the degree of divergence seen in genes not present in ORF4 (used to classify OsHV-1 microvariants), this work highlights the need to incorporate a more holistic genome-wide approach in understanding the role of OsHV-1 variants in ongoing mortality events. If only ORF4 were used to infer phylogenetic relationships, European µVar and Australian µVar-like samples would appear more closely related than indicated by the genome-wide analyses, thereby failing to capture the broader multi-clade structure of the sampled OsHV-1 genomic diversity.

East Asia has previously been proposed as a hotspot of OsHV-1 diversity and a possible geographic origin of OsHV-1 emergence (Mineur et al., 2015). Further, the existence of µVar-like genomes observed in Australia, New Zealand, and Japan (Jenkins et al., 2013; Renault et al., 2012; Shimahara et al., 2012), along with the low diversity of µVar genomes in Europe, support an East Asian origin hypothesis of the µVar variant (Mineur et al., 2015). The whole-genome analyses presented here provide a more detailed assessment of the relationships among the sampled Australian, New Zealand, European and Chinese genomes but do not independently establish their ancestral geographic origins. The clear phylogenetic clades observed at the whole-genome level suggest that Australian and New Zealand OsHV-1 form a grouping unique to both existing OsHV-1 variants in China and OsHV-1 µVar in Europe. Based on the level of genomic differentiation of Australian and New Zealand OsHV-1 from the OsHV-1 µVar, these sequences likely represent a distinct variant from µVar, despite similarities to µVar in the C-region of the genome. The nested placement of European microvariants within the C-region phylogeny is consistent with the previously proposed hypothesis of an exogenous origin for the emergence of µVar variants in Europe (Mineur et al., 2015). However, this relationship was not supported in the more information-rich phylogenies and no node within the C-region tree met the applied > 95% support threshold. Importantly, however, the European clade was not nested within a larger clade composed of samples from East Asia for phylogenies using larger portions of the genome; in fact, three diverged clades were observed one comprising of all Australian/New Zealand genomes, one comprising sequences Chinese, and one comprising of European genomes. The cause of this difference cannot be resolved using the currently available genomes. The limited whole-genome sampling from East and Southeast Asia may contribute to the absence of sampled lineages connecting the Chinese and European groups, but the existence and phylogenetic placement of such unsampled diversity cannot be assumed. Consequently, the lack of geographically representative whole-genome data currently precludes firm conclusions concerning the geographic origin or direction of spread of these lineages.

Although Australian and New Zealand samples sit within a monophyletic group, a clear subclade comprising samples collected from Tasmania in 2024 was observed. There are several possible causes for the observed Tasmanian 2024 subclade. For example, all 2024 Tasmanian samples were collected from wild oysters, compared to earlier years, which were farmed oysters. The heightened genetic differentiation may be the result of altered selection pressures on OsHV-1 within wild and farm-reared oysters, with OsHV-1 demonstrating rapid positive selection to adjust to specific host genetic backgrounds (Pelletier et al., 2025b). However, Tasmanian 2024 samples clustered outside of a monophyletic group with both samples from the initial New South Wales outbreak and other Tasmanian samples, suggesting they are different variants from those sequenced in Australia previously. One possible explanation for the 2024 Tasmanian subgroup is an independent introduction of a previously unsampled OsHV-1 lineage; however, this remains an untested hypothesis. All 2024 samples originated from wild oysters, whereas the earlier Australian samples were collected from farmed oysters, resulting in complete confounding among collection period, host source and phylogenetic grouping. Sequencing batch and genome quality also differ among these sample groups. The present dataset therefore cannot distinguish an independent introduction from host-associated selection, previously unsampled diversity circulating in wild oysters, or other temporal and methodological effects. The genomic distinction of the 2024 subgroup nevertheless warrants additional investigation because the emergence or introduction of divergent OsHV-1 lineages could have implications for breeding programs and aquaculture biosecurity. Resolving this question will require contemporaneous sampling of wild and farmed oysters, broader sampling across Australia and the Asia-Pacific region, and formal phylogeographic analysis.

Of the 123 unique genes described within OsHV-1 genomes, an estimated 70% have no known function (Davison et al., 2005; Segarra et al., 2014). The lack of a defined function for most genes in OsHV-1 limits the capacity to infer the importance of most mutations observed within Australian and New Zealand samples. Despite this limitation, coding-sequence variants were identified in eight genes with known or predicted functions. The functional effects of these variants were not assessed in the present study. Of particular note is the presence of twelve nonsynonymous mutations in the DNA polymerase gene, which is typically under extreme purifying selection due to its essential function (Morga et al., 2021). Although the effects of these substitutions are unknown, their occurrence in the DNA polymerase identifies them as candidates for future functional and selection analyses. Formal assessment of selection and polymerase activity would be required to determine whether any of these substitutions affect replication fidelity or viral mutation rate. Several genes are likely related to viral production, including both RR1 and RR2 subunits and the exonuclease; however, the effect of mutations in OsHV-1 genomes for these genes is unknown (Idowu et al., 1992; Mocarski, 2007). Finally, both IAP and the RING finger proteins are known to play a role in host immune evasion and pathogenicity (Boutell and Everett, 2013; Segarra et al., 2014). The IAP-encoding variants may warrant further investigation because mutations in IAP-encoding genes have also been reported in OsHV-1 variants associated with higher pathogenicity than the original OsHV-1 type (Martenot et al., 2013).

Mortality associated with OsHV-1 variants has declined over time in Australia (Cain et al., 2021), and several coding-sequence variants differed between samples from the initial New South Wales outbreak and later Tasmanian and South Australian samples. These included two variants in the DNA polymerase gene. Although viral genomic change represents one possible contributor to changing disease outcomes, the present data do not establish altered polymerase fidelity or a causal relationship between these variants and declining mortality. Host-side factors, including selective breeding for resistance, changes in host genetic composition, environmental conditions, and management practices, provide alternative and potentially interacting explanations. Controlled infection studies using characterised viral and host genotypes would be required to distinguish these possibilities.

## 5 Conclusions

This study presents the most extensive whole-genome characterisation of OsHV-1 μVar-like variants in Australia and New Zealand to date, revealing unexpected regional and temporal divergence among isolates sequenced. The identification of a genetically distinct Tasmanian subclade in 2024, may eitherrepresent a secondary incursion event or demonstrate previously unsampled genomic diversity within Australia, warranting further investigation. Critically, this work exposes a substantial gap in the availability of OsHV-1 whole-genome data globally and especially from East and Southeast Asia, regions central to OsHV-1 evolutionary history (Mineur et al., 2015). The lack of recent, comprehensive genomic surveillance across this region impedes efforts to accurately trace transmission pathways, assess virulence potential, and detect emerging pathogenic variants. Enhanced international sequencing efforts, particularly targeting underrepresented regions and wild oyster populations, are urgently needed to improve OsHV-1 risk assessment, inform breeding programs, and strengthen aquaculture biosecurity.

## Ethics approval and consent to participate

Not applicable.

## Funding

Funding for this work was provided by Commonwealth Scientific and Industrial Research Organisation (CSIRO), through the Infectious Animal Diseases and Zoonoses Program, and the Australian Government, Department of Agriculture Fisheries and Forestry (DAFF).

## CRediT authorship contribution statement

**James O’Dwyer:** Writing – review & editing, Writing – original draft, Methodology, Investigation, Formal analysis, Conceptualization. **Lachlan Coff:** Writing – review & editing, Writing – original draft, Validation, Methodology, Investigation, Formal analysis. **David Cummins:** Writing – review & editing, Writing – original draft, Methodology, Funding acquisition, Conceptualization. **Peter G. Mohr:** Project administration, Funding acquisition, Conceptualization. **Nicholas J. Moody:** Writing – review & editing, Writing – original draft, Project administration, Funding acquisition, Conceptualization.

## Declaration of competing interest

The authors declare no conflicts of interest.

## Data Availability

All whole genomes are deposited in NCBI under Accessions PZ564778- PZ564789. All OsHV-1 data for samples sequenced in this study are deposited in NCBI SRA under BioProject PRJNA1489751.
