# Supplementary material for: Whole genome characterisation of Australian and New Zealand OsHV-1 ‘μVar’ like variants over time
Source: Virus Res. 2026 Sep 9;373:199798. doi: 10.1016/j.virusres.2026.199798 (PMC13594741; doi:10.1016/j.virusres.2026.199798)
Supplement: Supplementary file 1 [file mmc1.docx]

**Supplementary materials III:** *Whole genome characterisation of Australian and New Zealand OsHV-1‘*μVar’ like variants over time.

James O’Dwyer^1^, Lachlan Coff^1^, David Cummins^1^, Peter Mohr^1^, Nick Moody^1^.

^1^*CSIRO Australian Centre for Disease Preparedness, East Geelong, Victoria 3220, Australia*

Per-base read depth was plotted across each assembled OsHV-1 genome to show the distribution of sequencing coverage and its relationship to the minimum depth used for final consensus generation.

**Figure S1.** Per-base read-depth profiles for the 12 Australian OsHV-1 genomes included in this study. Genome position is shown on the x-axis and mapped-read depth on the y-axis. The red dashed line indicates the minimum read depth of 5 used for final consensus generation. Y-axis scales differ among panels to display the coverage profile of each sample clearly.
