# Supplementary material for: Whole genome characterisation of Australian and New Zealand OsHV-1 ‘μVar’ like variants over time
Source: Virus Res. 2026 Sep 9;373:199798. doi: 10.1016/j.virusres.2026.199798 (PMC13594741; doi:10.1016/j.virusres.2026.199798)
Supplement: Supplementary file 2 [file mmc3.docx]

**Supplementary materials VII:** *Whole genome characterisation of Australian and New Zealand OsHV-1‘*μVar’ like variants over time.

James O’Dwyer^1^, Lachlan Coff^1^, David Cummins^1^, Peter Mohr^1^, Nick Moody^1^.

^1^*CSIRO Australian Centre for Disease Preparedness, East Geelong, Victoria 3220, Australia*

**Sensitivity test of highest coverage complete genes for phylogenetic inference**

A sensitivity analysis was conducted to determine whether the principal phylogenetic relationships recovered from the whole-genome alignment were retained when the analysis was restricted to complete coding sequences supported by a minimum read depth of 10. In this subset, a CDS was required to be both present in all assembled Australian and New Zealand genomes and had a minimum coverage of ≥10 across the entire coding region in every Australian and New Zealand genome. A total of 14 non-redundant CDS regions met the above criteria, comprising 9288bp in combined length (Table S1). The whole-genome baseline and DP≥10 complete-CDS trees were inferred from the same taxon set. Concordance was assessed using pairwise patristic-distance ordering (Spearman correlation), scaled branch-length agreement, quartet topology concordance, and recovery of splits supported at ≥80%.

| CDS | Length (bp)* |
| --- | --- |
| ORF1 | 447 |
| ORF2 | 504 |
| ORF3 | 444 |
| ORF4 | 1050 |
| ORF16 | 216 |
| ORF86 | 408 |
| ORF115 | 540 |
| ORF116 | 753 |
| ORF117 | 576 |
| ORF118 | 672 |
| ORF119 | 450 |
| ORF121 | 648 |
| ORF122 | 1155 |
| ORF124 | 1425 |

**Table S1.** CDS regions comprising the DP≥10 and all sample presence requirements for sensitivity testing. *Length is given as the length of the CDS in OsHV_NSW_2013_1.

The two analyses retained the Australian and New Zealand genomes as a consistent phylogenetic grouping, including the distinct cluster formed by the three Tasmanian genomes collected in 2024. Some external reference genomes changed relative position between the analyses, reflecting differences between the whole-genome dataset and the more stringently filtered, coding-sequence-only dataset. The quantitative comparison nevertheless showed strong overall concordance: Spearman r = 0.895 for patristic-distance ordering, scaled branch-length agreement = 0.760, quartet concordance = 0.850, and ≥80% supported-split precision = 0.900 (Table S2). Visual inspection shows limited deviation between the two phylograms generated (Figure S1).

| Metric | Value |
| --- | --- |
| Spearman correlation of patristic distances | 0.895 |
| Scaled branch-length agreement (1 - scaled NRMSE) | 0.760 |
| Quartet topology concordance | 0.850 |
| Supported-split precision at ≥80% | 0.900 |

**Table S2.** Quantitative concordance between the baseline whole-genome phylogeny and generated DP≥10 sensitivity testing tree.

**Figure S1.** Side-by-side comparison of the mid-rooted whole-genome maximum likelihood phylogram and the phylogeny restricted to complete CDS regions supported by a minimum read depth of 10. Both phylograms were generated using the HKY+F+I substitution model.
