# Supplementary material for: Whole genome characterisation of Australian and New Zealand OsHV-1 ‘μVar’ like variants over time
Source: Virus Res. 2026 Sep 9;373:199798. doi: 10.1016/j.virusres.2026.199798 (PMC13594741; doi:10.1016/j.virusres.2026.199798)
Supplement: Supplementary file 3 [file mmc4.docx]

**Supplementary materials VIII:** *Whole genome characterisation of Australian and New Zealand OsHV-1‘*μVar’ like variants over time.

James O’Dwyer^1^, Lachlan Coff^1^, David Cummins^1^, Peter Mohr^1^, Nick Moody^1^.

^1^*CSIRO Australian Centre for Disease Preparedness, East Geelong, Victoria 3220, Australia*

**Table S12.** Table of all transcriptionally relevant mutations in genes of known function observed in one or more OsHV-1 samples sequenced in this study. *Acronyms: Aus=Australia, NZ=New Zealand, Tas =Tasmania, SA=South Australia.

| Gene | Function | Reference | Alternate | Position (nucleotide position in gene) | Samples with mutation* |
| --- | --- | --- | --- | --- | --- |
| ORF100 | DNA polymerase | M | V | 7 | All Aus and NZ |
|  |  | D | N | 1306 | All Aus and NZ |
|  |  | L | V | 1768 | All Aus and NZ |
|  |  | A | S | 1093 | Tas and SA samples 2016-2019 |
|  |  | G | D | 2405 | Tas 2024 samples |
|  |  | P | S | 3367 | Tas 2024 samples |
|  |  | R | S | 3759 | All Aus and NZ |
|  |  | S | C | 4996 | All Aus and NZ |
|  |  | Y | H | 5023 | Tas and SA 2016-2019 samples |
|  |  | I | V | 5065 | All Aus and NZ plus MF509813.1 |
|  |  | T | M | 5285 | All Aus and NZ |
|  |  | T | I | 5372 | All Aus and NZ |
| ORF20 | RNR small subunit (R2) | I | S | 314 | All Aus and NZ |
|  |  | V | M | 898 | All Aus and NZ |
| ORF51 | RNR large subunit (R1) | S | F | 68 | Tas samples 2016-2019 collection |
|  |  | M | I | 375 | Tas samples 2024 collection |
|  |  | S | L | 695 | Tas samples 2024 collection |
| ORF95 | Exonuclease | K | T | 908 | All Aus and NZ |
| ORF42 | IAP protein | Q | - | 264-266 | All Aus and NZ |
| ORF87 | IAP protein | * | YFLHLQ* | 513-531 | All Aus and NZ |
| ORF9 | RING finger protein | R | S | 184 | All Aus and NZ plus MF509813.1 |
|  |  | T | S | 654 | Tas samples 2024 collection |
|  |  | A | V | 755 | All Aus and NZ |
|  |  | V | M | 771 | All Aus and NZ |
|  |  | E | A | 1460 | All Aus and NZ |
|  |  | - | E | 1492-1494 | All Aus and NZ |
| ORF41 | Envelope membrane protein | A | V | 1871 | All Aus and NZ |
