# Supplementary material for: Whole genome characterisation of Australian and New Zealand OsHV-1 ‘μVar’ like variants over time
Source: Virus Res. 2026 Sep 9;373:199798. doi: 10.1016/j.virusres.2026.199798 (PMC13594741; doi:10.1016/j.virusres.2026.199798)
Supplement: Supplementary file 4 [file mmc6.docx]

**Supplementary materials II:** *Whole genome characterisation of Australian and New Zealand OsHV-1‘*μVar’ like variants over time.

James O’Dwyer^1^, Lachlan Coff^1^, David Cummins^1^, Peter Mohr^1^, Nick Moody^1^.

^1^*CSIRO Australian Centre for Disease Preparedness, East Geelong, Victoria 3220, Australia*

**Positions of the designed probes mapped against the first round draft genome.**

**Figure S1**. Map of designed primer locations across the draft OsHV-1 genome OsHV_TAS_2016_1 (202,769 bp). Primer 8R, 9F and 9R are not shown because they correspond to regions of reference genome AY509253.2 rather than the draft genome. AY509253.2 coordinates: 8R = 191,517 bp; 9F = 198,984 to 196,196 bp; 9R = 196,196 to 198,984 bp.
