# Supplementary material for: Whole genome characterisation of Australian and New Zealand OsHV-1 ‘μVar’ like variants over time
Source: Virus Res. 2026 Sep 9;373:199798. doi: 10.1016/j.virusres.2026.199798 (PMC13594741; doi:10.1016/j.virusres.2026.199798)
Supplement: Supplementary file 5 [file mmc8.docx]

**Supplementary materials V:** *Whole genome characterisation of Australian and New Zealand OsHV-1‘*μVar’ like variants over time.

James O’Dwyer^1^, Lachlan Coff^1^, David Cummins^1^, Peter Mohr^1^, Nick Moody^1^.

^1^*CSIRO Australian Centre for Disease Preparedness, East Geelong, Victoria 3220, Australia*

**GARD analysis of impact of putative recombination events on phylogenetic signal.**

Phylogenetic concordance was evaluated using four complementary metric classes after the baseline and partition trees were pruned to identical taxon sets. First, normalized Robinson–Foulds (RF) distance quantified the proportion of exact bipartition splits differing between trees (Robinson & Foulds, 1981). Second, Spearman correlation of all pairwise patristic distances assessed whether the relative ordering of between-sample evolutionary distances was retained (Steel & Penny, 1993). Third, partition patristic distances were rescaled to the baseline by least-squares proportional scaling and the remaining discrepancy was summarized as normalized root-mean-square error (NRMSE), providing a branch-length-sensitive comparison related to path-distance and branch-score approaches (Kuhner & Felsenstein, 1994; Steel & Penny, 1993). Finally, quartet concordance measured the proportion of resolved four-taxon subsets supporting the same topology in both trees (Steel & Penny, 1993). Supported-split precision and recall at the joint ≥80% SH-aLRT/ultrafast-bootstrap threshold were also reported to distinguish strongly supported contradictory splits from reduced resolution in shorter partitions. Precision describes the proportion of supported partition splits also present in the baseline, whereas recall describes the proportion of supported baseline splits recovered by the partition.

Across the nine evaluable partitions, Spearman correlations were high (0.812–0.970) and quartet concordance ranged from 0.870 to 0.997. Supported-split precision at the ≥80% threshold ranged from 0.733 to 1.000, indicating that most strongly supported relationships in the partition trees were also present in the baseline. Lower recall in several short partitions primarily reflected reduced resolution rather than extensive supported contradiction. Partition 8 was not evaluated because only one sequence remained after missing-data filtering.

| Partition | Alignment bp | Taxa | nRF | Spearman r | Scaled NRMSE | Quartet concord. | ≥80% split P / R |
| --- | --- | --- | --- | --- | --- | --- | --- |
| 1 | 1–20,253 | 41 | 0.526 | 0.940 | 0.201 | 0.884 | 0.933 / 0.538 |
| 2 | 20,254–46,357 | 41 | 0.553 | 0.955 | 0.178 | 0.996 | 1.000 / 0.577 |
| 3 | 46,358–67,872 | 41 | 0.605 | 0.854 | 0.441 | 0.947 | 0.846 / 0.423 |
| 4 | 67,873–89,549 | 41 | 0.579 | 0.870 | 0.404 | 0.870 | 0.733 / 0.423 |
| 5 | 89,550–178,578 | 41 | 0.474 | 0.970 | 0.183 | 0.974 | 0.900 / 0.692 |
| 6 | 178,579–182,545 | 38 | 0.800 | 0.874 | 0.247 | 0.887 | 0.800 / 0.174 |
| 7 | 182,546–186,115 | 41 | 0.421 | 0.848 | 0.334 | 0.952 | 0.846 / 0.423 |
| 9 | 188,878–216,228 | 41 | 0.500 | 0.890 | 0.243 | 0.996 | 0.938 / 0.577 |
| 10 | 216,229–217,884 | 24 | 0.667 | 0.812 | 0.659 | 0.997 | 1.000 / 0.143 |

**Table S1.** Quantitative concordance between the baseline whole-genome phylogeny and each evaluable GARD partition tree.

nRF, normalized Robinson–Foulds distance; NRMSE, root-mean-square error after least-squares scaling of partition patristic distances to the baseline; P, supported-split precision; R, supported-split recall. Partition 8 was excluded because insufficient taxa remained after missing-data filtering (only one geonome was present in the partition).

**Figure S1.** Multi-metric comparison of the baseline whole-genome phylogeny with each evaluable GARD partition tree.

Panels show Spearman correlation of pairwise patristic distances, scaled branch-length discrepancy expressed as NRMSE relative to the mean baseline distance, quartet concordance, and supported-split precision and recall at the joint ≥80% SH-aLRT/ultrafast-bootstrap threshold. Partition 8 was not evaluated because insufficient taxa remained after missing-data filtering. High Spearman correlation, quartet concordance and supported-split precision indicate broad preservation of phylogenetic relationships, while lower recall identifies partitions with reduced resolution.

**Figure S2.** Baseline mid-rooted whole-genome maximum likelihood phylogram of OsHV-1 genomes sequenced/assembled in this study (excluding OsHV_NSW_2010_1, OsHV_TAS_2024_1, and OsHV_TAS_2024_3) and all available OsHV-1 whole genomes sequences available from the NCBI. The phylogram was generated using the HKY+F+I substitution model. Bootstrap and SH-aLRT support values are shown per branch in the format (Bootstrap/SH-aLRT)

**Figure S3.** Partition one (bp 1-20,253 of multi sequence alignment) of mid-rooted phylogram of OsHV-1 genomes sequenced/assembled in this study (excluding OsHV_NSW_2010_1, OsHV_TAS_2024_1, and OsHV_TAS_2024_3) and all available OsHV-1 whole genomes sequences available from the NCBI. The phylogram was generated using the substitution model K3Pu+F+I. Ultra-fast bootstrap support values are shown per branch.

**Figure S4.** Partition two (bp 20,254-46,357 of multi sequence alignment) of mid-rooted phylogram of OsHV-1 genomes sequenced/assembled in this study (excluding OsHV_NSW_2010_1, OsHV_TAS_2024_1, and OsHV_TAS_2024_3) and all available OsHV-1 whole genomes sequences available from the NCBI. The phylogram was generated using the substitution model HKY+F+I. Ultra-fast bootstrap support values are shown per branch.

**Figure S5.** Partition three (bp 46,358-67,872 of multi sequence alignment) of mid-rooted phylogram of OsHV-1 genomes sequenced/assembled in this study (excluding OsHV_NSW_2010_1, OsHV_TAS_2024_1, and OsHV_TAS_2024_3) and all available OsHV-1 whole genomes sequences available from the NCBI. The phylogram was generated using the substitution model HKY+F+I. Ultra-fast bootstrap support values are shown per branch.

**Figure S6.** Partition four (bp 67,873-89,549 of multi sequence alignment) of mid-rooted phylogram of OsHV-1 genomes sequenced/assembled in this study (excluding OsHV_NSW_2010_1, OsHV_TAS_2024_1, and OsHV_TAS_2024_3) and all available OsHV-1 whole genomes sequences available from the NCBI. The phylogram was generated using the substitution model HKY+F+I. Ultra-fast bootstrap support values are shown per branch.

**Figure S7.** Partition five (bp 89,550-178,578 of multi sequence alignment) of mid-rooted phylogram of OsHV-1 genomes sequenced/assembled in this study (excluding OsHV_NSW_2010_1, OsHV_TAS_2024_1, and OsHV_TAS_2024_3) and all available OsHV-1 whole genomes sequences available from the NCBI. The phylogram was generated using the substitution model HKY+F+I. Ultra-fast bootstrap support values are shown per branch.

**Figure S8.** Partition six (bp 178,579-182,545 of multi sequence alignment) of mid-rooted phylogram of OsHV-1 genomes sequenced/assembled in this study (excluding OsHV_NSW_2010_1, OsHV_TAS_2024_1, and OsHV_TAS_2024_3) and all available OsHV-1 whole genomes sequences available from the NCBI. The phylogram was generated using the substitution model HKY+F. Ultra-fast bootstrap support values are shown per branch.

**Figure S9.** Partition seven (bp 182,546-186,115 of multi sequence alignment) of mid-rooted phylogram of OsHV-1 genomes sequenced/assembled in this study (excluding OsHV_NSW_2010_1, OsHV_TAS_2024_1, and OsHV_TAS_2024_3) and all available OsHV-1 whole genomes sequences available from the NCBI. The phylogram was generated using the substitution model TPM3u+F+I. Ultra-fast bootstrap support values are shown per branch.

**Figure S10.** Partition nine (bp 188,878-216,228 of multi sequence alignment) of mid-rooted phylogram of OsHV-1 genomes sequenced/assembled in this study (excluding OsHV_NSW_2010_1, OsHV_TAS_2024_1, and OsHV_TAS_2024_3) and all available OsHV-1 whole genomes sequences available from the NCBI. The phylogram was generated using the substitution model HKY+F+I. Ultra-fast bootstrap support values are shown per branch.

**Figure S11.** Partition ten (bp 216,229-217,884 of multi sequence alignment) of mid-rooted phylogram of OsHV-1 genomes sequenced/assembled in this study (excluding OsHV_NSW_2010_1, OsHV_TAS_2024_1, and OsHV_TAS_2024_3) and all available OsHV-1 whole genomes sequences available from the NCBI. The phylogram was generated using the substitution model HKY+F+I. Ultra-fast bootstrap support values are shown per branch. Missing samples are indicative of samples which were entirely gaps for this partition.

**References**

Kuhner, M. K., & Felsenstein, J. (1994). A simulation comparison of phylogeny algorithms under equal and unequal evolutionary rates. *Molecular Biology and Evolution*, *11*(3), 459–468. https://doi.org/10.1093/oxfordjournals.molbev.a040126

Robinson, D. F., & Foulds, L. R. (1981). Comparison of phylogenetic trees. *Mathematical Biosciences*, *53*(1), 131–147. https://doi.org/10.1016/0025-5564(81)90043-2

Steel, M. A., & Penny, D. (1993). Distributions of Tree Comparison Metrics—Some New Results. *Systematic Biology*, *42*(2), 126–141. https://doi.org/10.1093/sysbio/42.2.126
